# Construction of the graph genomes of *Takifugu* provides novel insights into the genomic mechanisms of population structure and migratory traits

**DOI:** 10.1186/s12915-025-02296-7

**Published:** 2025-07-01

**Authors:** Rui-shi He, Rong Zhao, Jun-jie Lin, Yan Li, Xiang-zhu Kong, Jin-xuan Xu, Jing-hang Wu, Xing-jiang Bu, Yong-jun Zhang, Yang Sun

**Affiliations:** 1 Key Laboratory for Conservation and Use of Important Biological Resources of Anhui Province, Anhui Provincial Key Laboratory of Molecular Enzymology and Mechanism of Major Diseases, College of Life Sciences, Anhui Normal University, Wuhu, China; 2 Laboratory for Biology of Plant Diseases and Insect Pests, Institute of Plant Protection, Chinese Academy of Agricultural Sciences, Beijing, China

**Keywords:** *Takifugu*, Synthetic-based pan-genome, Graph pan-genome, PAV, SV

## Abstract

**Background:**

The genus *Takifugu* includes highly valued fish species known for their delicate flavor, making them popular in multiple countries. However, many species from this genus face significant threats. In order to better understand the genetic diversity and evolutionary dynamics of *Takifugu*, a syntelog-based pan-genome and graph genome were constructed using the data of seven *Takifugu* species.

**Results:**

The analysis of 28,085 syntelog groups (SGs) composed of protein-coding genes revealed that only 57.3% of the SGs were shared among all individuals, whereas the remaining genes presented presence-absence variation (PAV) across the seven genomes. Using the graph genome as a reference, a population of 160 *Takifugu* individuals was analyzed, from which 20,133,471 SNPs, 4,606,141 Indels, and 152,200 SVs were identified. The gene flow analysis revealed directional gene flow from *Takifugu bimaculatus* and *Takifugu flavidus* to *Takifugu oblongus*. Notably, a 51-bp insertion in the *ABCB9* gene differed significantly in frequency between the two migratory populations, suggesting the potential role of this gene in the migratory behavior of these species. Additionally, the expression profiles from 13 tissues or organs (brain, gallbladder, gill, gonad, heart, kidney, liver, muscle, pituitary, skin, spleen, stomach, and swim bladder) revealed a unique expression pattern in the liver, with the tissue-specific genes exhibiting evolutionary conservation to varying degrees. The highest proportion of core genes was found in the pituitary, whereas the lowest was found in the spleen.

**Conclusions:**

This study provides comprehensive genomic resources that enhance the understanding of the genetic diversity and evolutionary dynamics of *Takifugu* species. The findings offer insights for research on both breeding and conservation of *Takifugu*.

**Supplementary Information:**

The online version contains supplementary material available at 10.1186/s12915-025-02296-7.

## Background

The genus *Takifugu* belongs to the phylum Chordata, class Actinopterygii, order Tetraodontiformes, and family Tetraodontidae. *Takifugu* fish, commonly known as pufferfish, comprises about 25 species that are distributed widely in the northwest Pacific, including the coastal waters of China, Korea, and Japan [49, 73]. These fish are renowned for their ability to inflate and the presence of tetrodotoxin (TTX) in their internal organs. In addition, these fish have highly palatable flesh, due to which *Takifugu* species have been farmed commercially in several Asian countries, including China, Korea, and Japan, in recent years [73]. *Takifugu rubripes* and *Takifugu xanthopterus* are economically important fish species in China. Although *Takifugu* species share close phylogenetic relationships, their morphology, coloration, and toxicity levels are significantly different across different species. Two distinct migratory behaviors are observed within the *Takifugu* genus – long-distance upstream migration, as demonstrated by *Takifugu obscurus* and *Takifugu ocellatus*, and short-distance seasonal migration, represented by *T. rubripes*; these behaviors reflect species-specific adaptations to salinity [29]. Despite the phenotypic differences between populations, natural hybridization events have been reported among *Takifugu* species. For example, the hybrids of *T. rubripes* and *Takifugu porphyreus* were found to have relatively low concentrations of TTX [51]. These hybridization events contribute to increasing the genetic diversity within *Takifugu* populations and provide valuable resources for breeding programs aimed at improving the different strains of *Takifugu* and increasing their economic value.

However, despite their economic importance, certain *Takifugu* species are encountering significant conservation challenges. *Takifugu chinensis*, for example, is a commercially valuable species, which has been listed as critically endangered under the A2bd criteria due to an estimated population decline of more than 80% expected over the next three generations, primarily due to overfishing and habitat degradation. This species dwells in the sandy and muddy seabeds of the East China Sea and Yellow Sea at depths of 5–150 m, but a dramatic decline in its catch rates has occurred since the 1970 s [22]. Similarly, other species, such as *Takifugu flavidus*, *T. rubripes*, and *T. ocellatus,* are listed as near-threatened, further emphasizing the vulnerability of various *Takifugu* species [49].

With advancements in high-throughput sequencing technologies, the genomes of various *Takifugu* species have been published in recent years [20, 67, 75, 77], which has laid the foundation for population-level analyses of *Takifugu*. Zhang et al. identified 16 osmoregulation-related genes through the resequencing analyses of five *Takifugu* species [70]. Similarly, Zhou et al. conducted a genome-wide association study (GWAS) based on the resequencing data from *Takifugu* and identified 122,573 single nucleotide polymorphisms (SNPs), among which 9 were significantly associated with growth traits and involved 17 genes [78]. However, most population genomic resequencing studies on *Takifugu* to date have relied on the mapping of short reads to a single reference genome to identify the genetic variations, including SNPs, insertions and deletions (InDels), and structural variations (SVs). However, this approach presents limitations in the study of population diversity. Moreover, population analyses based on a single reference genome do not fully represent the entire genetic diversity of a species [15]. In recent years, several studies on pan-genomes, particularly graph pan-genomes, have been reported [17, 24, 27, 30, 44]. Compared to traditional linear reference genomes, graph pan-genomes illustrate genomic structural diversity in the form of a graph rather than being constrained by a single reference genome. Therefore, these pan-genomes can simultaneously depict multiple types of variations, such as insertions, deletions, inversions, and duplications, providing a better representation of complex genomic variations. In the case of species with high genetic diversity, constructing graph pan-genomes can significantly increase the detection of genetic variants, reduce reference bias, and offer a further accurate and comprehensive framework for analyzing genetic diversity among populations [76].

In this study, a graph pan-genome was constructed for *Takifugu* using seven chromosome-level genomes, and the graph pan-genome was employed as a reference to genotype 160 *Takifugu* resequencing samples. The study identified a total of 20,133,471 SNPs, 4,606,141 Indels, and 15,200 high-quality SVs. Using allele frequency differences and XP-CLR analysis, SVs and SNPs that could influence the migratory behavior of *Takifugu* were identified. Additionally, a comprehensive transcriptomic analysis was performed for multiple *Takifugu* tissues, revealing tissue-specific gene expression patterns and immune gene expression profiles specific to three tissues (spleen, gill, and kidney) under different conditions. The findings provided valuable resources and novel insights for *Takifugu* genome research, offering important implications for understanding the salinity adaptability of this genus, which would aid in the conservation of endangered *Takifugu* species and guide a variety of improvement and breeding programs.

## Results

### Reassembly and reannotation of the 7 *Takifugu* genomes

**Fig. 1:**
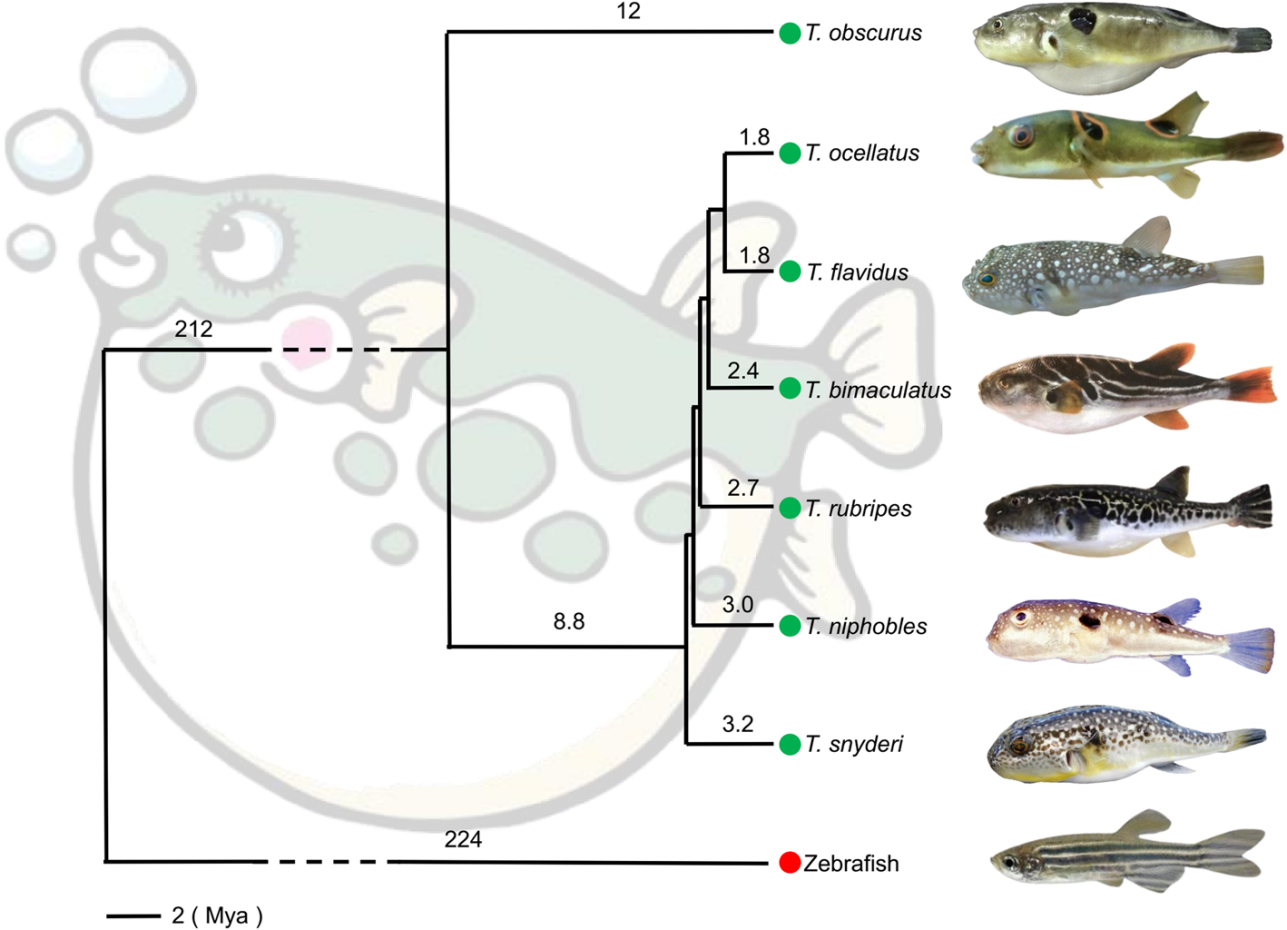
Maximum likelihood phylogenetic tree constructed using 1,200 orthologous single-copy genes, with zebrafish serving as the outgroup and seven *Takifugu* species as the focus

In order to construct a graphical pan-genome, it is essential to reassemble and reannotate genomes to ensure consistency across datasets. Therefore, in this study, genomic data from seven *Takifugu* individuals were collected, including six chromosome-level genomes (*T. flavidus*, *T. rubripes*, *T. bimaculatus*, and three species with unavailable chromosome-level assemblies: *T. snyderi*, *T. obscurus*, and *T. niphobles*) and one scaffold-level genome (*T. ocellatus*). The genomes of *T. snyderi*, *T. obscurus*, and *T. niphobles* lacked chromosome-level assemblies and, therefore, reassembly was conducted and the genome of *T. flavidus*, which had the highest BUSCO, was used as a reference to correct and reannotate gene structures in the other six genomes. The results revealed differences among the seven *Takifugu* species in terms of genome size, gene number, and the quantity and distribution of transposable elements (TEs). The genome sizes ranged from 350 to 393 Mb, and the BUSCO completeness scores ranged between 95.2% and 97.7%, indicating high-quality assembly and correction of the genomes in this study. The annotation of repetitive sequences identified 34.3 Mb to 52.9 Mb of the sequences in these genomes as TE regions, whereas 30.6 Mb to 65.7 Mb were tandem repeat sequences. The gene structure annotation of the non-repetitive regions revealed 19,918 to 21,067 protein-coding genes (Table 1). A phylogenetic tree was constructed using 1,200 orthologous single-copy genes from the seven species to reveal the evolutionary relationships of these species (Fig. 1). *T. obscurus* diverged from the other six *Takifugu* species about 12 million years ago (Mya), while *T. niphobles* and *T. snyderi* were the most closely related species that diverged about 3.2 Mya. The assembly and analysis of these genomic datasets laid a foundation for future research on the evolutionary history of the *Takifugu* genus and the identification of the candidate genes associated with phenotypic traits.

**Table 1:** Summary of the genome assembly and annotation of *Takifugu*

Sample	Assembly Size (Mb)	Geng Number	TE Number	TE Size (Mb)	TRF Size (Mb)	Busco (%)
*T. niphobles*	377.5	20,345	127,529	52.7	36.4	97.2
*T. bimaculatus*	404.4	21,853	135,615	52.9	38.6	95.2
*T. flavidus*	366.3	20,389	99,034	44.5	30.6	96.7
*T. obscurus*	365.1	20,308	82,654	36.0	32.2	97.7
*T. ocellatus*	375.6	19,918	101,220	42.2	65.7	97.4
*T. rubripes*	384.1	21,067	106,942	41.8	36.1	96.8
*T. snyderi*	360.1	20,283	76,594	34.3	33.4	97.0

### Construction of the syntelog-based pan-genome

**Fig. 2:**
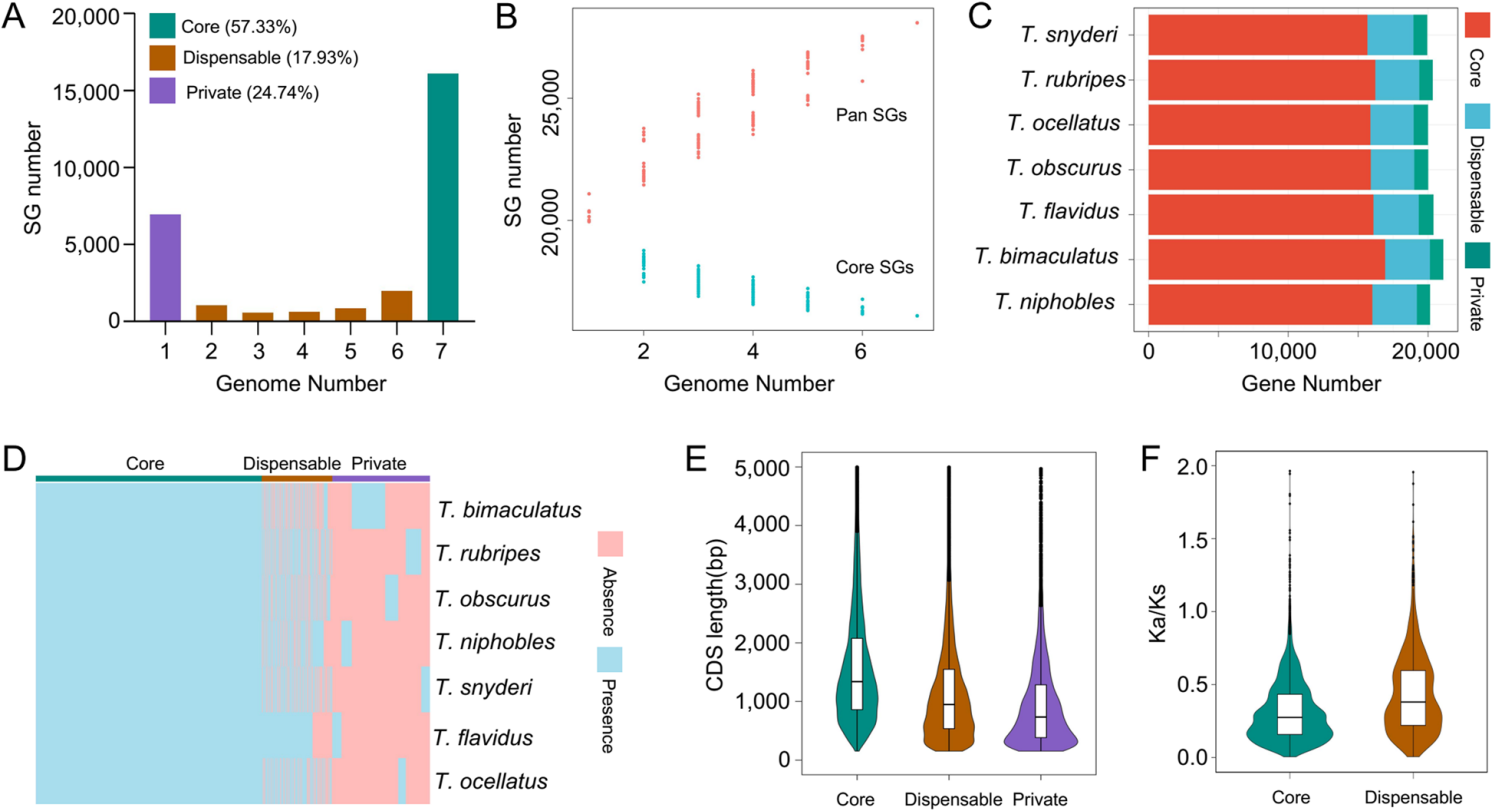
Composition and characteristics of the syntelog-based pan-genome. **A** The number of different types of SGs present across the (1–7) *Takifugu* genomes. **B** Trends in the total number of SGs in the pan-genome and the number of core SGs with increasing number of genomes. As one genome was added randomly, the pan SGs and core SGs were recalculated. This process was repeated 100 times. **C** The numbers of the three types of SGs (core, dispensable, and private) in the genomes of different *Takifugu* species. **D** PAV heatmap of the SGs across the *Takifugu* genome. **E** Distribution of CDS lengths for genes in the three types of SGs. **F** Ka/Ks values of the genes in the core and dispensable SGs

By constructing a syntelog-based pan-genome, the PAVs of the genes across different species can be revealed, which is crucial for understanding the genetic diversity and adaptability of species. In this study, pairwise comparisons of the seven *Takifugu* genomes were performed to identify SGs, and accordingly, a syntelog-based *Takifugu* pan-genome was constructed. The syntelog-based pan-genome included 28,085 SGs, of which 57.3% (16,100) were core SGs, 17.9% (5,036) were dispensable SGs, and 24.7% (6,949) were private SGs (Fig. 2A). As the number of genomes increased, the number of core SGs gradually decreased, whereas the number of pan SGs continued to increase (Fig. 2B). Among the seven *Takifugu* genomes, the core SGs accounted for 76.8% to 80.7%, dispensable SGs accounted for 12.2% to 17.8%, and private SGs accounted for 3.6% to 10.9% (Fig. 2C and D), indicating the conservation of genes varies across genomes and that private SGs contributed to the genetic diversity of the pan-genome. Additionally, the CDS length of the core SGs was significantly longer than that of dispensable and private SGs (Fig. 2E), suggesting that the core SGs may have more stable functions and evolutionary conservation, whereas dispensable and private SGs are probably involved in rapid adaptation or species-specific functions. Further analysis of the Ka/Ks values of different genes in these SGs revealed that dispensable SGs presented significantly higher Ka/Ks values than the core SGs (Fig. 2F), indicating that dispensable SGs are under stronger positive selection and may play a critical role in environmental adaptation and species differentiation.

**Fig. 3:**
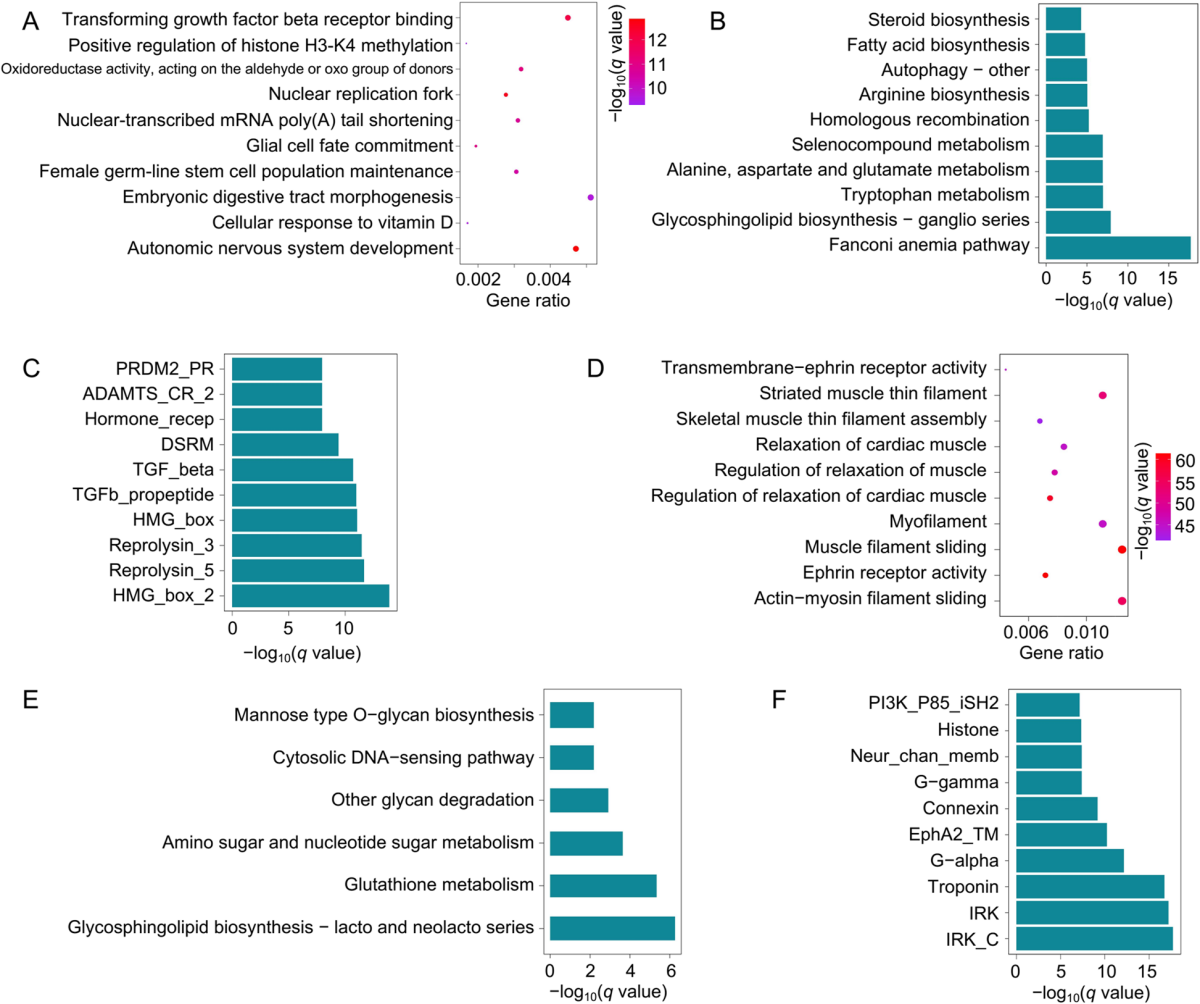
The GO enrichment (**A**), KEGG enrichment (**B**), and Pfam domain enrichment (**C**) of genes in the core SGs in the Takifugu pan-genome. The GO enrichment (**D**), KEGG enrichment (**E**), and Pfam domain enrichment (**F**) of genes in the dispensable SGs in the *Takifugu* pan-genome

In order to further elucidate the functions of core SGs, dispensable SGs, and private SGs, enrichment analyses were conducted on these three types of SGs. The enrichment analysis of the core SGs revealed that these genes were enriched primarily in functions related to basic life processes, such as autonomic nervous system development, embryonic digestive tract morphogenesis, glycosphingolipid biosynthesis—ganglio series, tryptophan metabolism, and high mobility group (HMG) proteins (Fig. 3A-C). This finding highlighted the essential biological processes and conserved functions of the core SGs, providing novel insights into their role in the fundamental life activities of different *Takifugu* species. In contrast, dispensable genes were enriched in functions related to the phenotypic differences between species. For example, the GO and Pfam enrichment analyses of these genes revealed associations with muscle contraction and relaxation, including the regulation of muscle relaxation, transmembrane ephrin receptor activity, troponin, IRK, and histones (Fig. 3D and F). KEGG enrichment analysis revealed involvement in glutathione metabolism (Fig. 3E), and the glutathione-S-transferase family (GST) involved in this pathway has been linked to paralytic shellfish toxins (PST), a neurotoxin similar to TTX [36]. These results suggest that the dispensable SGs likely play a significant role in phenotypic divergence, toxin metabolism, and the regulation of muscle and neural functions across different species.

### Construction of the graph pan-genome and SV map

**Fig. 4:**
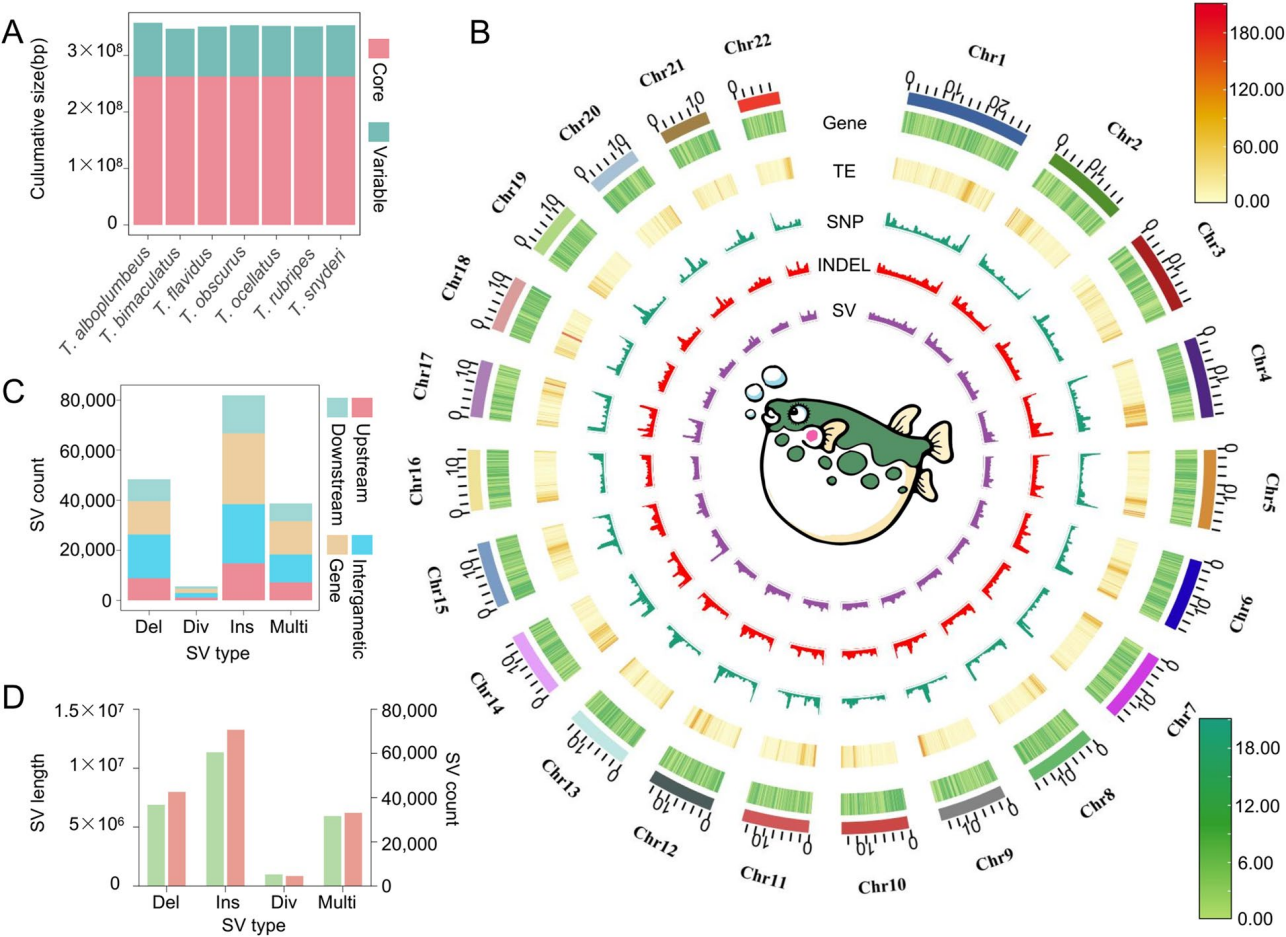
Genomic features of different *Takifugu* species. **A** The sizes of the core and variable nodes in each genome. **B** Reference genome assembly characteristics and the variation landscape (SNPs, indels, and SV calling based on 160 resequenced data samples). **C** The number of SVs in the gene intervals, 5 kb upstream and downstream of the genes, and intergenic regions. **D** The number and length distribution of the different types of SVs in the *Takifugu* graph pan-genome. Green represents the SV length, and pink represents the SV count

The graph pan-genome overcomes the limitations of individual genomes and offers significant advantages in detecting complex variations, such as SVs. In this study, the *T. flavidus* genome was used as a ‘backbone’ and compared to the genomes of six other *Takifugu* species to construct the *Takifugu* graph pan-genome. The graph pan-genome had a size of 604 Mb and was composed of 2,553,597 bubbles, including the 657,224 core nodes shared by all genomes and 1,896,373 variable nodes. The total length of the core nodes was 262.5 Mb, while the length distribution of the variable nodes across different genomes ranged from 84.6 to 95.2 Mb, accounting for about 25% of the total genome size (Fig. 4A). These results indicated that, as *Takifugu* species evolved and adapted to different environmental pressures, structural differences appeared in their genomes. This is reflected in the constructed graph pan-genome.

The graph pan-genome revealed extensive SVs within the *Takifugu* genomes, which may be associated with the biological traits and environmental adaptations of the different species of this genus. In order to explore the distribution of these SVs in a broader *Takifugu* population, 160 *Takifugu* whole-genome sequencing samples were genotyped using Vg software. After removing the genotypes with a missing rate of > 0.3 or minor allele frequency (MAF) < 0.05, a total of 20,133,471 SNPs, 4,606,141 Indels, and 152,200 SVs were identified (Fig. 4B). Among the different types of SVs, most were located within the genes or in 5 kb upstream or downstream regions, suggesting that these SVs may have significant impacts on gene function and expression (Fig. 4C). On the basis of the allele type, SVs can be categorized into biallelic (insertions, deletions, and divergent alleles) and multiallelic variations. Insertions were the most abundant type, whereas multiallelic variants were the least common revealed in this study (Fig. 4D). These findings demonstrated the presence of extensive SVs across the *Takifugu* species, and this information may be used for further research into the phenotypic differences across *Takifugu* species.

### Population analysis of *Takifugu*

**Fig. 5:**
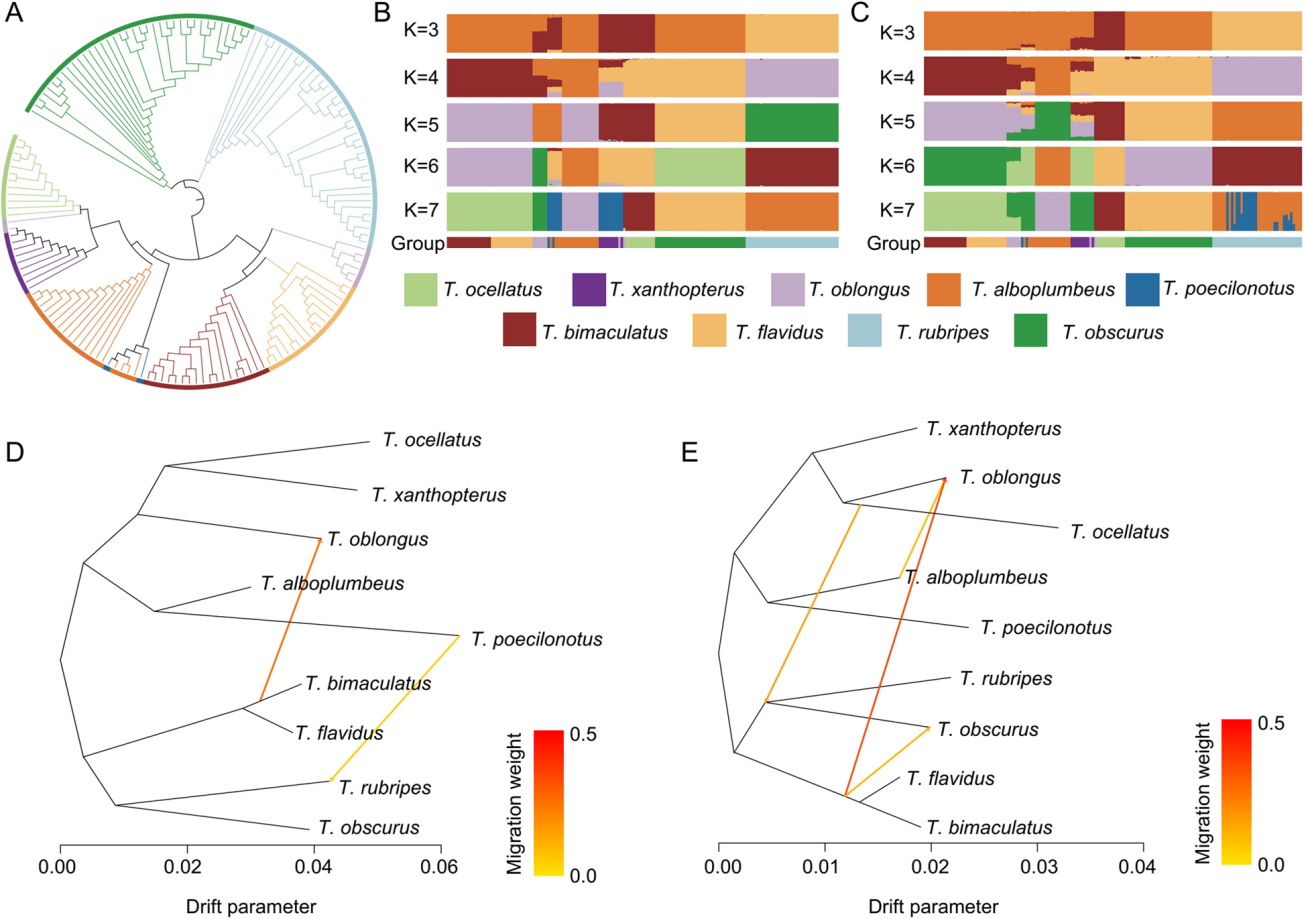
Population analysis of different *Takifugu* species. **A** Phylogenetic tree of *Takifugu* species constructed on the basis of population genomic SNPs (**B**-**C**) SNP-based (**B**) and SV-based (**C**) population structure analysis. The species colors depicted in the phylogenetic tree correspond to those in the population structure analysis, with the color for each species labeled below the population structure plot. **D**, **E** Gene flow analysis conducted on the basis of population genomic SNP (**D**) and SV (**E**) profiles

The phylogenetic relationships and genetic diversity across *Takifugu* species have been a research focus for a long time [43]. Therefore, a population analysis was conducted for 9 *Takifugu* species on the basis of SNPs and SVs. The phylogenetic tree constructed from the SNPs indicated that *T. bimaculatus* and *T. flavidus* formed a sister group, with both species closely related to *T. oblongus* (Fig. 5A). Although most populations clustered into a single branch on the evolutionary tree, *T. alboplumbeus* and *T. poecilonotus* were interspersed along the same branch, suggesting a close phylogenetic relationship between these two species and a potential gene flow between them. The ADMIXTURE population structure analysis conducted based on both SNPs and SVs revealed that at K = 4, the population structure aligned most closely with the phylogenetic tree. Moreover, both SNP- and SV-based population structure analyses revealed consistent compositions for *T. bimaculatus* and *T. flavidus* at K = 2–7 (Fig. 5B and C). Population-based SNP and SV analyses can reveal different gene flow phenomena, associated with the distinct types of polymorphic markers (SNPs or SVs) present in the genomes of different individuals. The gene flow analysis based on SNPs and SVs in this study revealed more gene exchange among *Takifugu* species than among the SNPs, suggesting that SVs are more effective genomic markers for detecting gene flow than SNPs. Notably, both SNP- and SV-based gene flow analyses revealed gene flow from *T. bimaculatus* and *T. flavidus* to *T. oblongus* (Fig. 5D and E). ABBA/BABA analysis further supports this conclusion (Additional file 1: Fig. S1). These findings enhance the understanding of the genetic interactions between *Takifugu* species and provide a more nuanced perspective on their evolutionary relationships and genetic exchange mechanisms.

### Key variations influencing the migratory behavior of *Takifugu*

**Fig. 6:**
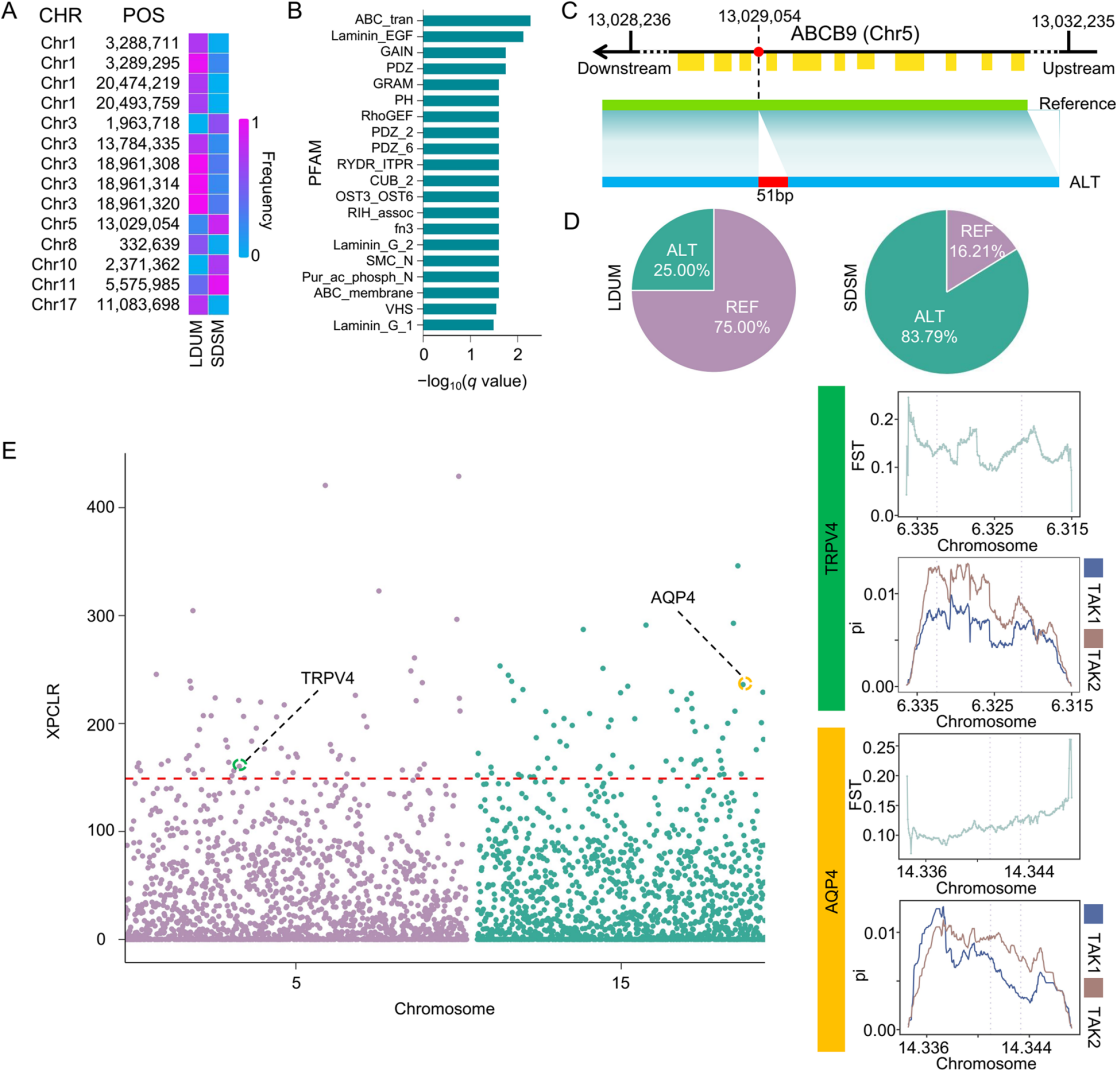
Important variations affecting the migration behavior of *Takifugu*. **A** Heatmap of the SV allele frequency differences (> 0.5) between the different migratory populations located in the gene regions or in the ± 2 kb upstream/downstream regions of the genes enriched in the ABC transporter pathway. **B** Pfam enrichment analysis of the genes with gene regions or ± 2 kb upstream/downstream regions located in the SVs with allele frequency differences > 0.5 between the different migratory populations. **C** Diagram of the SVs occurring in *ABCB9* (*Takifugu_flavidus05314*) between the two migratory populations. **D** Frequency of the SVs occurring in *ABCB9* in the two migratory populations. **E** Selection signal analysis of the two migratory populations. The red dashed line indicates the significance threshold, and the two circled points indicate the genes located in the selection signal regions

Compared to short-distance seasonally migrating (SDSM) anadromous fish, long-distance upstream migrating (LDUM) fish must undergo long-distance overwintering migrations, during which the latter faces various biotic and abiotic stresses, including salinity and temperature changes [59, 65]. In this study, on the basis of SNP and SV data from different *Takifugu* populations, a genetic differentiation analysis between two migratory types (LDUM: *T. obscurus* and *T. ocellatus*; SDSM: *T. bimaculatus*, *T. flavidus*, *T. alboplumbeus*, *T. poecilonotus*, T*. xanthopterus*, *T. oblongus*, and *T. rubripes*) was conducted [65]. The results revealed 2,806 SVs with allele frequency differences greater than 0.5 between the two migratory types, which were distributed within the gene regions or in 2 kb upstream and downstream regions of 879 genes. The enrichment analysis of these genes revealed significant enrichment in the KEGG ABC transporter pathway, whereas the Pfam domain analysis revealed enrichment in the ABC_tran, Laminin_EGF, and ABC_membrane domains (Fig. 6A and B, Additional file 2: Table 1). These findings suggested that the genes related to ABC transporters may play important roles in the adaptation process of different migratory types. Notably, ABCB9 was a key gene among these enriched candidates, which is located in the major histocompatibility complex (MHC) region. Since long-distance migratory fish traverse more complex water environments, they inevitably encounter diverse pathogenic microorganisms [40]. Therefore, variations in the MHC region genes may help the fish to adapt to pathogens in different environments. The results of this study revealed a 51 bp insertion in the *ABCB9* gene (allele frequency: LDUM 0.25/SDSM 0.84), which may be one of the key SVs influencing the immune function of the *Takifugu* species (Fig. 6C and D).

The migration of *Takifugu* fish is an important reproductive behavior, with both *T. obscurus* and *T. ocellatus* observed to migrate to the Yangtze River for spawning during the breeding season [21]. This process requires adaptation to the changes in salinity. In this study, a whole-genome selection signal scan of the SNPs in the population was performed within a 1 kb sliding window, which identified 1,542 regions exhibiting selection signals (Fig. 4E). Among these genes, 2,381 were located in the selection signal regions (Additional file 3: Table 2). These genes included 38 genes involved in the calcium signaling pathway. The calcium signaling pathway is believed to be associated with maintaining osmotic pressure and regulating the intracellular calcium concentration, among various other vital physiological processes [7]. The interaction between the *aquaporin-4* (*AQP4*) and transient receptor potential *vanilloid 4* (*TRPV4*) genes, which are located in these selection signal regions (*AQP4*: *Takifugu_flavidus14661* and *TRPV4*: *Takifugu_flavidus04898* located on Chr15:14,340,001–14370000 and Chr5: 6,310,001–6330000), is critical for sperm motility under hyperosmotic conditions. Moreover, the Fst and Pi values for the *AQP4* and *TRPV4* gene regions and their 2 kb upstream and downstream regions were calculated for the two populations. The results revealed significant genetic differentiation between the two populations for these genes (Fig. 6E), suggesting that these genes may play important roles in adapting to different salinity environments during the reproductive process of the migrating populations. In summary, these findings provided novel insights and resources for further research on deciphering the biological mechanisms underlying *Takifugu* migration.

### Transcriptomic characteristics of multiple tissues and organs in the* Takifugu* pan-genome and populations

**Fig. 7:**
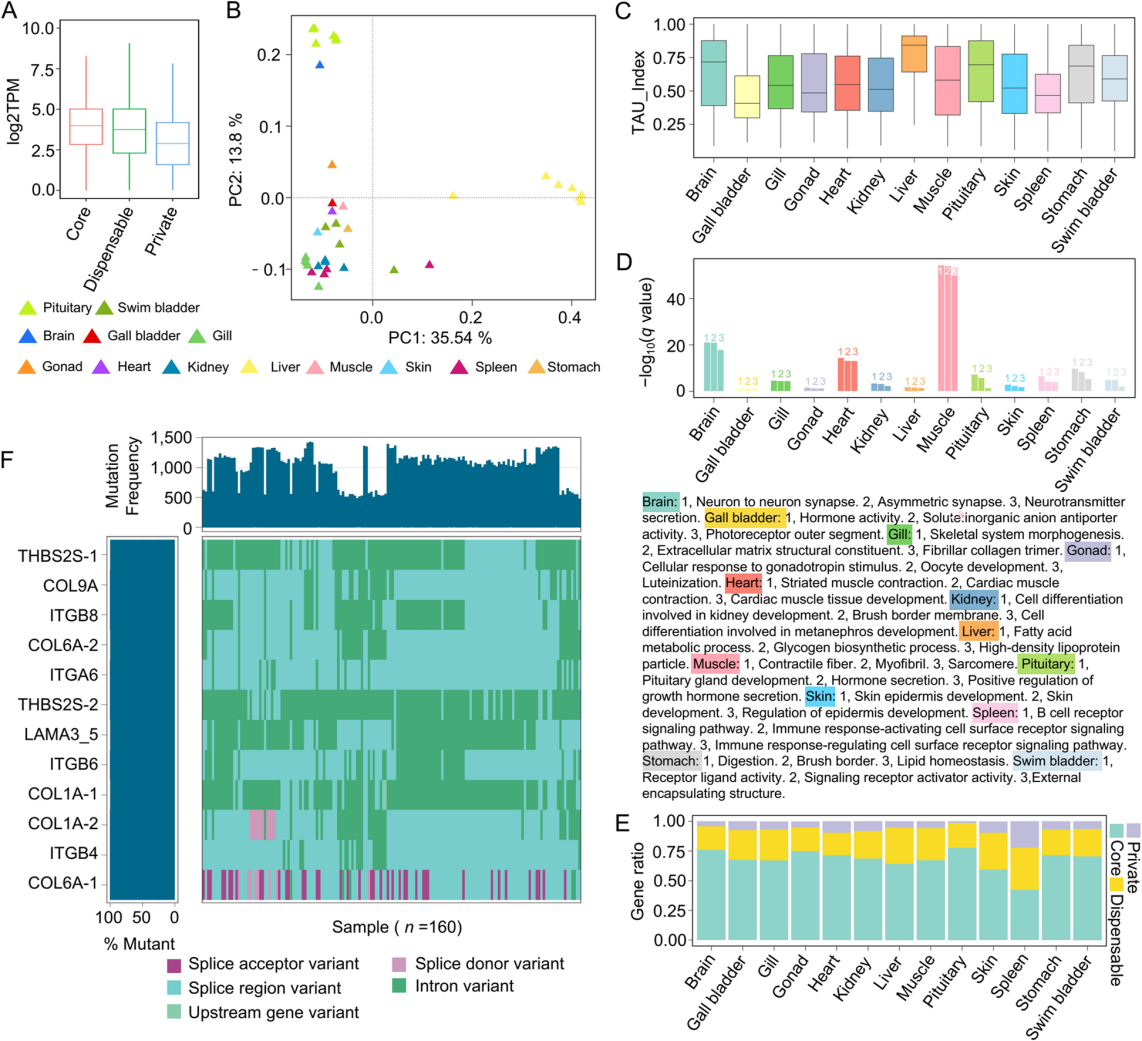
Multi-tissue transcriptomic atlas of *Takifugu*. **A** Expression level distribution of genes with different PAV types. **B** PCA of the transcriptomic data. **C** TAU distribution of genes across various organs or tissues. **D** GO enrichment analysis of genes with tissue- or organ-specific expression. **E** The proportion of tissue-specific genes expressed with different PAV types across various organs or tissues. **F** Mutation burden waterfall plot of the DEGs enriched in the ECM-receptor interaction pathway

Although previous studies have revealed variations and the related genes associated with selection through population genomics and pangenomic analyses, the lack of deeper integration of the gene expression data has prevented the comprehensive understanding of gene functions. Transcriptome analysis is crucial for studying animal growth, development, and environmental adaptation. Therefore, in this study, the RNA-seq data from 13 different tissues and organs of *Takifugu* were quantitatively analyzed. Compared to private genes, core and dispensable genes presented higher expression levels, with core genes having slightly higher expressions than the dispensable genes. These findings suggested that core genes may play a more important role in maintaining essential biological functions (Fig. 7A). The PCA of the transcriptome revealed significant differences in the estimated gene expression levels in the liver along the PC1 axis compared to those for the other tissues (Fig. 7B), indicating that the liver presented a distinct gene expression pattern. Furthermore, the tissue-specific analysis revealed that the genes in the liver presented the highest TAU values (Fig. 7C), suggesting a relatively high level of expression specificity in the liver, which is consistent with the PCA results. Functional enrichment analysis of the tissue-specific genes revealed that their functions closely aligned with the physiological roles of their respective tissues and organs. For example, genes specifically expressed in the brain were enriched in the GO terms such as “neuron to neuron synapse” and “neurotransmitter secretion”, whereas genes specifically expressed in the muscle were enriched in the GO terms such as “contractile fiber” and “myofibril”. In addition, the distribution of the PAV types of tissue-specific genes varied across different tissues. For example, the proportion of core genes was the highest in the pituitary and lowest in the spleen (Fig. 7E), suggesting significant differences in the conservation of tissue-specific genes across different tissues.

Immune-related genes play critical roles in the adaptation of *Takifugu* to various environmental pressures and the enhancement of disease resistance. Investigating the variations in these genes within populations, therefore, provides deeper insights into the survival strategies of *T. rubripes* across diverse ecological environments, particularly revealing how long-distance migrating individuals adapt and survive while traversing multiple aquatic and microbial environments. In this study, a differential expression analysis was performed using the RNA-seq data from 25 samples derived from spleen, gill, and kidney tissues across 2 immune-related projects. A total of 441 DEGs were identified (Additional file 4: Table 3). The KEGG enrichment analysis of these DEGs revealed 12 genes enriched in the ECM-receptor interaction pathway (Additional file 5: Table 4), which has been implicated in immune response processes [53]. By integrating the SNP data from population genomics, the genetic variations in the 12 genes enriched in the ECM-receptor interaction pathway were determined (Fig. 7F, Additional file 6: Table 5). Interestingly, each gene exhibited different types of mutations across every individual sample, suggesting that these genes were under strong selection pressure within the population. Notably, intron variants were the most prevalent type of mutation observed in these genes. In addition to intron variants, a significant number of splice-related mutations, including splice acceptor, splice region, and splice donor variants, were identified. These splice-related mutations could alter the splicing process of pre-mRNAs, thereby affecting protein structure and ultimately influencing the phenotypes. These diverse genetic variation types and their potential functional impacts could be important clues for further investigations into the regulatory mechanisms and functions of immune-related genes.

## Discussion

Genome-wide analysis conducted using genome sequencing is crucial for studying genetic diversity across different species. With advancements in third-generation sequencing technologies, research on pan-genomes has been successfully implemented in various plants and animals, including humans [14, 45], yaks [24], sheep [28], rice [44], and soybeans [31]. However, most of the current research on the genomes of aquatic species has been focused primarily on single-reference genome studies [6, 56, 69], which severely hinders the development of aquaculture breeding programs. In this study, a syntelog-based pan-genome and graph-based pan-genome were constructed for the *Takifugu* genus using the genomes of seven different *Takifugu* species and the resequencing data from 160 individuals. According to the PAV classification of the pan-genome, more than half of the SGs (57.3%) were classified as core SGs. This high proportion of core SGs is likely due to the relatively conserved nature of vertebrate genomes and the limitations of the genome assembly scale. Nonetheless, 5,036 dispensable SGs (accounting for 7.9% of the pan-genome genes) and 6,949 private SGs (accounting for 24.7% of the pan-genome genes) were identified. A functional enrichment analysis of these genes revealed that the functions of core SGs are associated mainly with fundamental life activities, whereas dispensable SGs are involved primarily in metabolism, biosynthesis, and muscle regulation, suggesting that dispensable SGs may contribute to the phenotypic differences between different species (Fig. 3). Furthermore, dispensable SGs presented lower Ka/Ks ratios than core SGs, indicating that dispensable SGs experienced greater selective pressure during evolution (Fig. 2F). Similar findings have been reported for the pan-genomes of other species, such as soybean [31] and water [68, 69, 72], in which core genes are more conserved than dispensable genes. This underscores the importance of studying pan-genomes for understanding the genetic basis of phenotypic diversity.

Previous studies have shown that constructing a graph-based pan-genome allows for a more comprehensive identification of high-quality genetic variations [57]. So far, a large number of high-quality variations have been identified in many species through the construction of graph-based pan-genomes. For example, the rice graph pan-genome was identified with an average of 24,469 SVs per accession [41], the tomato graph pan-genome was identified with 17,898,731 SNPs, 1,499,161 Indels, and 195,957 SVs [76], and the yak graph pan-genome was identified with 1,048,639 SNPs and 610,921 SVs [30]. The graph pan-genome constructed in this study contained 20,133,471 SNPs, 4,606,141 Indels, and 15,200 SVs. These high-quality SNP and SV datasets were then utilized for population structure and evolutionary analysis. The results of both population structure analysis and phylogenetic tree constructed based on the SNPs and SVs indicated that the nine *Takifugu* species could be divided into four groups (Fig. 5A-C), among which *T. bimaculatus* and *T. flavidus* presented similar population structures. This is consistent with the findings of Liu et al. [29], who constructed a phylogenetic tree based on 4DTV and mitochondrial genetic variation sites. This consistency demonstrates that graph pan-genome analyses can accurately reveal the evolutionary relationships and population differentiation mechanisms among *Takifugu* species.

In recent years, an increasing number of studies have confirmed that SVs provide more information on variation than SNPs when studying breeding and population differentiation [28, 76]. This study revealed that SVs were associated with more gene flow events among *Takifugu* species. For example, the gene flow from *T. rubripes* and *T. obscurus* to *T. oblongus* occurred once each. The gene flow from *T. alboplumbeus* to *T. oblongus* also occurred once (Fig. 5D and E). Furthermore, the high-quality SNPs and SVs generated from the resequencing data and graph-based pan-genomes enabled a more comprehensive identification of the selection patterns among different populations. In this study, differences in the SV frequencies of several members of the *ABC* gene family were observed between two different migratory populations (Fig. 6A-D). The structure and function of the* ABC* gene family are highly conserved in vertebrates and are widely involved in critical physiological processes such as the transport of cellular material and stress responses [11, 62]. The findings of this study suggest that differences in the SV frequencies among populations may lead to changes in the ABC gene family, which influences the migration-related traits between the two populations. Additionally, the selection signal analysis based on SNPs revealed a water channel protein-encoding gene (*AQP4*) that is strongly associated with osmotic regulation (Fig. 6E). *AQP4* has been demonstrated to play a critical role in osmotic regulation in various aquatic animals [11, 33, 58]. These findings not only uncover the genetic basis of adaptive population evolution but also serve as valuable resources for subsequent functional analyses and molecular mechanism studies on this genus.

Using integrated transcriptomics analysis, a better understanding of the relationships between different genetic variations and gene expression across the pan-genome was obtained. The TAU index calculations and the functional enrichment of tissue-specific genes revealed that organ-specific genes are often related to the functions performed by these organs. For example, the genes specifically expressed in the brain (such as trio, brsk1, and nrxn1) were involved in neurotransmitter secretion in the brain and, therefore, play important roles in regulating growth hormone secretion in fish [37]. Moreover, genes that are specifically expressed in the brain, gonad, heart, and pituitary were predominantly the core genes identified in this study (Fig. 7E), indicating the conserved nature of these genes and their minimal selective pressure during evolution. On the other hand, in tissues such as the skin, liver, and spleen, dispensable and private genes constituted a relatively large proportion of tissue-specific genes, and these genes were enriched primarily in immune response, detoxification, and skin development functions. Studies have shown that different *Takifugu* species exhibit variations in skin color, body size, and TTX (tetrodotoxin) content [71, 78]. This suggests that these genes may be associated with phenotypic differences across populations. In summary, these results provide novel insights into the genetic differences between different populations and offer resources and references for *Takifugu* breeding and conservation.

## Conclusions

This study constructed a pangenome and graph genome for the *Takifugu* genus, revealing extensive gene PAV, gene flow, and key structural variants in migration-related genes (e.g., *ABCB9*), shedding light on ecological adaptation. A multi-tissue expression atlas showed contrasting evolutionary conservation among organ-specific genes, with pituitary genes being highly conserved and spleen genes less so. These results advance our understanding of fish genome diversity and provide genomic resources for *Takifugu* breeding.

## Methods

### Data acquisition

The genomic data for seven *Takifugu* species were obtained from NCBI (https://www.ncbi.nlm.nih.gov/), including chromosome-level genomes for *T. flavidus*, *T. rubripes*, and *T. bimaculatus*, as well as read data of non-chromosome-level assemblies for *T. ocellatus* and read data for *T. snyderi*, *T. obscurus* and *T. niphobles*, for which chromosome-level assemblies are unavailable (Additional file 7: Table 6). Whole-genome sequencing (WGS) data were sourced from two projects (PRJNA522329 and PRJNA638440), encompassing 160 samples from nine species – *T. rubripes*, *T. ocellatus*, *T. flavidus*, *T. bimaculatus*, *T. obscurus*, *T. snyderi*, *Takifugu alboplumbeus*, *T. oblongus*, and *Takifugu xanthopterus* (Additional file 8: Table 7).

### Genome assembly and annotation

The three species for which the assemblies of chromosome-level genomes were not available (*T. flavidus*, *T. rubripes*, and *T. bimaculatus*) were subjected to de novo assembly using NextDenovo (v2.5.2) [19] with read_cutoff = 10 kb and genome_size = 400 Mb. The assembled contigs were polished using Pilon [54], resulting in three draft genomes. On the basis of the BUSCO (v5.8.2) [46] evaluation of the three chromosome-level genomes (*T. flavidus*, *T. bimaculatus*, and *T. rubripes*), *T. flavidus*, which presented the highest BUSCO score was selected as the reference genome (Additional file 9: Table 8). Minimap2 (v2.28) [25] was then used with default parameters to align the genomes of the remaining six species to the reference genome. Subsequently, RagTag (v2.1.0) [1] was used with “scaffold” and “correct” parameters to scaffold and reorient these genomes relative to the reference, resulting in the final assembly of the seven *Takifugu* genomes. All the genomes were reevaluated using BUSCO to ensure quality and completeness.

Gene structure annotation was performed for each genome using an integrated approach that combined three methods: de novo gene prediction, RNA-Seq evidence, and protein homology [18]. Repetitive sequences were identified using RepeatMasker (v4.1.7) [52] with the parameters “-nolow -no_is -norna” and RepeatModeler (v2.0.5) [12] with a custom repeat library, whereas tandem repeats were detected using Tandem Repeats Finder (TRF) (v4.09) [2]. The identified repetitive sequences were masked for the subsequent annotation analysis. Next, ab initio gene prediction was conducted using Augustus (v3.5.0) [48], with the genome and protein-coding genes of *T. flavidus* used as the training set. Predictions were guided using MAKER2 with default parameters. For transcriptomic evidence, RNA-Seq data from 36 samples were collected (Additional file 10: Table 9). The RNA-Seq reads were filtered using Fastp [5] (default parameters) to remove the adapters and low-quality sequences. Transcripts were then assembled using SOAPdenovo-Trans (v1.0.5) [63], and redundancy was reduced using CD-HIT (v4.8.1) [13]. SOAPdenovo-Trans was run with the parameters “max_rd_len = 50, rd_len_cutoff = 45, and avg_ins = 200”, whereas CD-HIT was used with default settings. Finally, gene prediction was integrated using the MAKER2 pipeline (v2.31.10), which combined the evidence from de novo prediction, RNA-Seq, and protein homology. The protein-coding genes shorter than 50 amino acids and with AED > 0.5 were filtered out, resulting in a high-confidence set of gene models.

The transposons in each genome were annotated using EDTA (v2.0.0) [35] with default settings. The functional annotation of the genes in each genome was performed as follows: Gene Ontology (GO) terms were assigned using EggNOG-mapper (v2.1.2) [3] with the Actinopteri database; KEGG pathway annotations were conducted using KOBAS (v2.0) [61], Pfam domains were identified using HMMER hmmsearch (v3.4) [9].

### Phylogenetic analysis and divergence time estimation

The GRCz11 version of the zebrafish genome was downloaded from the NCBI database. Orthologous genes in this genome were identified using Orthofinder (v2.5.5) [10] with the"-d"parameter based on the CDSs of zebrafish and the seven *Takifugu* species. Considering zebrafish as an outgroup, a species phylogenetic tree was constructed using CDS sequences of single-copy orthologous genes via CASTER (Chao [68, 69, 72]) with default parameters (including 1000 local block bootstraps and the F84 nucleotide substitution model). Branch lengths of the phylogenetic tree were calculated using IQ-TREE [34], applying the -g option to constrain the topology to CASTER's tree output while optimizing only branch lengths. The estimation of species divergence times was performed with r8s (v1.8.1) [42].

### Syntelog-based pan-genome construction

The syntenic relationships among the seven genomes were determined and used to construct a syntelog-based pan-genome. First, pairwise whole-genome alignments of the sequences from the seven genomes were performed using Diamond [60]. The alignment results were filtered and only the best hits were retained. Next, DAGchainer [16] was employed to detect the syntenic genomic regions and syntelogs. After the syntenic relationships of the genes across genomes were determined, SynPan (https://github.com/dongyawu/PangenomeEvolution) [60] was used to iteratively merge the pairwise syntenic information, with *T. flavidus* used as the initial framework. If a gene from an additional genome was syntenic to a gene in the previously merged pan-genome, it was assigned to an existing SG. If the gene was not syntenic to any gene in the merged iterative pan-genome, a new SG was created. The seven genomes were merged as a syntelog-based pan-genome. On the basis of the PAVs of SGs across genomes, the SGs were further classified into three categories: core (present in all species), dispensable (present in 2–6 species), and private (present in only one species). The PAV types of genes were consistent with their corresponding SGs.

Enrichment analysis of the different types of SGs was performed using clusterProfiler in the R package [66]. The Ka and Ks values of different gene pairs among different types of SGs were calculated using KAKS_CALCULATOR [55].

### Construction of a graph pan-genome and SVs and SNP calling

First, using *T. flavidus* as the reference framework, the remaining six genomes were incrementally integrated into assemblies and used to construct a multi-assembly graph using MiniGraph (v0.20) [26] with the “-cxasm” and “-call” parameters. SVs were derived from the graph-based genome using the bubble-popping algorithm in gfatools (v0.5) [26] with “gfabed” parameter. Each bubble represented an SV, which was defined by the start and end nodes of the reference sequence, as well as the paths traversing these nodes. The SVs were classified as biallelic if a bubble contained two paths and as multiallelic if the bubble contained more than two paths. Finally, PanPop [74] was used to genotype the 160 resequencing samples, yielding high-confidence SNPs, indels, and SVs.

### Population analysis

Principal component analysis (PCA) and population structure analysis based on the identified SVs and SNPs were performed using GCTA (v1.94.1) [64] and ADMIXTURE (v1.3.0) [50], respectively. In order to construct the phylogenetic tree based on SNPs, nucleotide sequences were first extracted from the SNP file and used for the construction of a tree using CASTER [68]. The tree file was visualized using iTOL (https://itol.embl.de/upload.cgi). The selective signal analysis based on SNPs was conducted using xpclr (v1.1.2) [4], with each chromosome analyzed independently and divided into non-overlapping 10 kb windows. Treemix (with default parameters) was utilized to investigate gene flow among different populations [39]. Additionally, ABBA/BABA analysis was conducted using ABABAwindows.py (https://github.com/simonhmartin/genomics_general/blob/master/ABBABABAwindows.py), with *T. rubripes* serving as the outgroup. The average XP-CLR score was calculated for each window. Regions with the top 20% of the XP-CLR likelihood scores were merged into a single window if adjacent or separated by just one window. The maximum average XP-CLR likelihood score among these merged regions was assigned to the new window. The regions with the top 5% of the XP-CLR scores were considered to exhibit strong selection signals.

### Transcriptome analysis

A total of 54 RNA-seq datasets from 13 tissues of *Takifugu* (brain, gallbladder, gill, gonad, heart, kidney, liver, muscle, pituitary, skin, spleen, stomach, and swim bladder) were collected (Additional file 11: Table 10). The raw transcriptome data were first processed using fastp (v0.23.4) to remove the low-quality sequences and adapter contamination. The cleaned data were then mapped to the *T. flavidus* genome using HISAT2 with “–dta” parameters. Afterward, the BAM files were sorted with SAMtools (v1.13) [8], and the gene expression levels were quantified using StringTie (v2.2.3) [38]. The differentially expressed genes (DEGs) were identified using the R package DESeq2 [32].

For tissue-specific analysis, the tissue specificity index (TAU) of the genes in each tissue was calculated using the following formula:

$$Tau=\frac{\sum_{i=1}^n\left(1-\frac{x_i}{{\underset{1\leq i\leq n}{\text{max}}x_i}}\right)}{n-1}$$

Here, n is the number of tissues, x denotes the median TPM expression of the gene in the tissue, and i represents a specific tissue. The TAU value ranges from 0 to 1, where 0 indicates broad expression across tissues and 1 represents highly tissue-specific expression. Genes with a TAU value > 0.8 were considered tissue-specific and subjected to a GO enrichment analysis using the R package clusterProfiler, with the GO annotations of the *T. flavidus* genome serving as the background.

In order to illustrate the variation in the immune-related genes within the population, a differential expression analysis was performed using DESeq2 for samples with *Vibrio harveyi* infection and poly(I:C), and the results were compared with those of their respective controls. For genes enriched in the ECM-receptor interaction pathway, the SNPs located within these gene regions were extracted and annotated using Variant Effect Predictor (VEP, v99) [23]. Finally, a waterfall plot was generated using the R package GenVisR [47].

## Supplementary Information

Additional file 1: Fig. S1. Box plot illustrating the D statistics from the two populations.

Additional file 2: Table 1. Results of the KEGG enrichment analysis of genes associated with the SVs showing frequency differences between the two migratory populations.

Additional file 3: Table 2. Genes located in the XP-CLR selection signal regions identified in this study.

Additional file 4: Table 3. Differentially expressed immune-related genes identified in this study.

Additional file 5: Table 4. Results of the KEGG enrichment analysis of immune-related DEGs.

Additional file 6: Table 5. Variation information of the immune-related DEGs enriched in the ECM-receptor interaction pathway.

Additional file 7: Table 6. An overview of the Takifugu genome assembly and sequencing information.

Additional file 8: Table 7. Resequencing information of the 160 samples analyzed in this study.

Additional file 9: Table 8. Results of the BUSCO evaluation of the six reassembled and corrected *Takifugu* genomes.

Additional file 10: Table 9. The RNA-seq data used for gene structure annotation in this study.

Additional file 11: Table 10. The RNA-seq data used for the transcriptomic characteristic analysis of the pan-genome in this study.

## Data Availability

Genomic data for seven Takifugu species were obtained from NCBI (https://www.ncbi.nlm.nih.gov/), including chromosome-level genomes for T. flavidus, T. rubripes, and T. bimaculatus, as well as read data of non-chromosome-level assemblies for T. ocellatus, and read data for T. snyderi, T. obscurus and T. niphobles, for which chromosome-level assemblies are unavailable (Additional file 7: Table 6). Whole Genome Sequencing (WGS) data were sourced from two projects (PRJNA522329 and PRJNA638440), encompassing 160 samples from nine species (T. rubripes, T. ocellatus, T. flavidus, T. bimaculatus, T. obscurus, T. snyderi, T. alboplumbeus, T. oblongus, and T. xanthopterus) (Additional file 8: Table 7). We provide the graphical pan-genome information of Takifugu (10.6084/m9.figshare.27717891). The genome folder contains the genome sequence of Takifugu, the gff folder stores the gene annotation information of Takifugu, and the graph_genome folder contains the graphical pan-genome sequence.

## Abbreviations

SG: Syntelog groups
PAV: Presence-absence variation
SNP: Single nucleotide polymorphism
SV: Structural variation
GWAS: Genome-wide association study
InDel: Insertions and deletion
TRF: Tandem repeats finder
PCA: Principal component analysis
TAU: Tissue specificity index
TE: Transposable element
Mya: Million years ago
CDS: Coding sequence
Ka: Nonsynonymous substitution rate
Ks: Synonymous substitution rate
HMG: High mobility group
GO: Gene ontology
Pfam: Protein family
KEGG: Kyoto encyclopedia of genes and genomes
GST: Glutathione-S-transferase family
PST: Paralytic shellfish toxins
MAF: Minor allele frequency
SDSM: Short-distance seasonal migration
LDUM: Long distance upstream migration
MHC: Major histocompatibility complex
AQP4: Aquaporin-4
TRPV4: Receptor potential vanilloid 4
4DTV: Four-fold degenerate sites
TTX: Tetrodotoxin
