# Supplementary figures and images for: Construction of the graph genomes of Takifugu provides novel insights into the genomic mechanisms of population structure and migratory traits
Source: BMC Biol. 2025 Jul 1;23:195. doi: 10.1186/s12915-025-02296-7 (PMC12220038; doi:10.1186/s12915-025-02296-7)

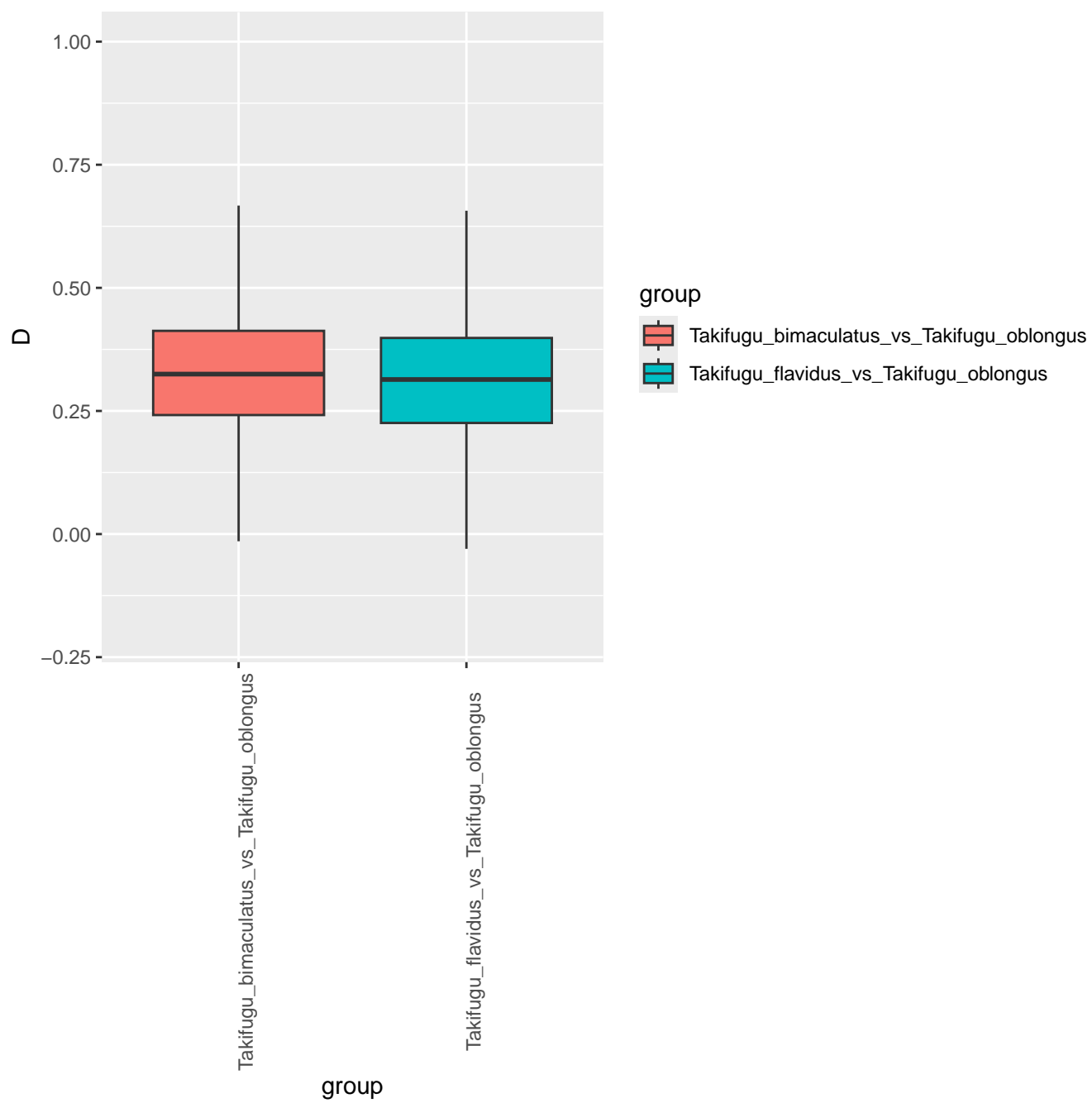

Supplement: Supplementary file 3 — Additional file 3: Table 2. Genes located in the XP-CLR selection signal regions identified in this study. [file 12915_2025_2296_MOESM3_ESM.pdf]
